# A multi-regions discrete-time epidemic model with a travel-blocking vicinity optimal control approach on patches

**DOI:** 10.1186/s13662-017-1168-4

**Published:** 2017-04-26

**Authors:** Omar Zakary, Mostafa Rachik, Ilias Elmouki, Samih Lazaiz

**Affiliations:** 1 Laboratory of Analysis, Modeling and Simulation (LAMS), Department of Mathematics and Computer Science, Hassan II University of Casablanca, Sidi Othman, BP 7955, Casablanca, Morocco; 2 Laboratory of Algebra, Analysis and Applications (L3A), Department of Mathematics and Computer Science, Hassan II University of Casablanca, Sidi Othman, BP 7955, Casablanca, Morocco

**Keywords:** multi-regions model, SIR epidemic model, discrete-time model, optimal control, vicinity, travel-blocking

## Abstract

We study, in this paper, infection dynamics when an epidemic emerges to many regions which are connected with their neighbors by any kind of anthropological movement. For this, we devise a multi-regions discrete-time model with the three classical SIR compartments, describing the spatial-temporal behaviors of homogenous susceptible, infected and removed populations. We suppose a large geographical domain, presented by a grid of colored cells, to exhibit at each instant *i* the spatial propagation of an epidemic which affects its different parts or sub-domains that we call here cells or regions. In order to minimize the number of infected individuals in some regions, we suggest an optimal control approach based on a travel-blocking vicinity strategy which aims to control a group of cells, or a patch, by restricting movements of infected people coming from its neighboring cells. We apply a discrete version of Pontryagin’s maximum principle to state the necessary conditions and characterization of the travel-blocking optimal controls. We provide cellular simulations based on discrete progressive-regressive iterative schemes associated with the obtained multi-points boundary value problems. For illustrating the modeling and optimal control approaches, we consider an example of 100 regions.

## Introduction

### Main references and description of the problem

In 1927, Kermack and McKendrick devised the Susceptible-Infected-Removed (SIR) model which has presented an interesting contribution to the mathematical theory of epidemics [1]. The mathematical SIR model is in the form of three compartments: susceptible, infected or removed. Susceptible populations are healthy and do not carry the epidemic but can contract it from infected individuals which carry the infection and can pass it to susceptible hosts, while the removed people are no longer infected and acquire immunity from future contagion.

**Figure 1:**
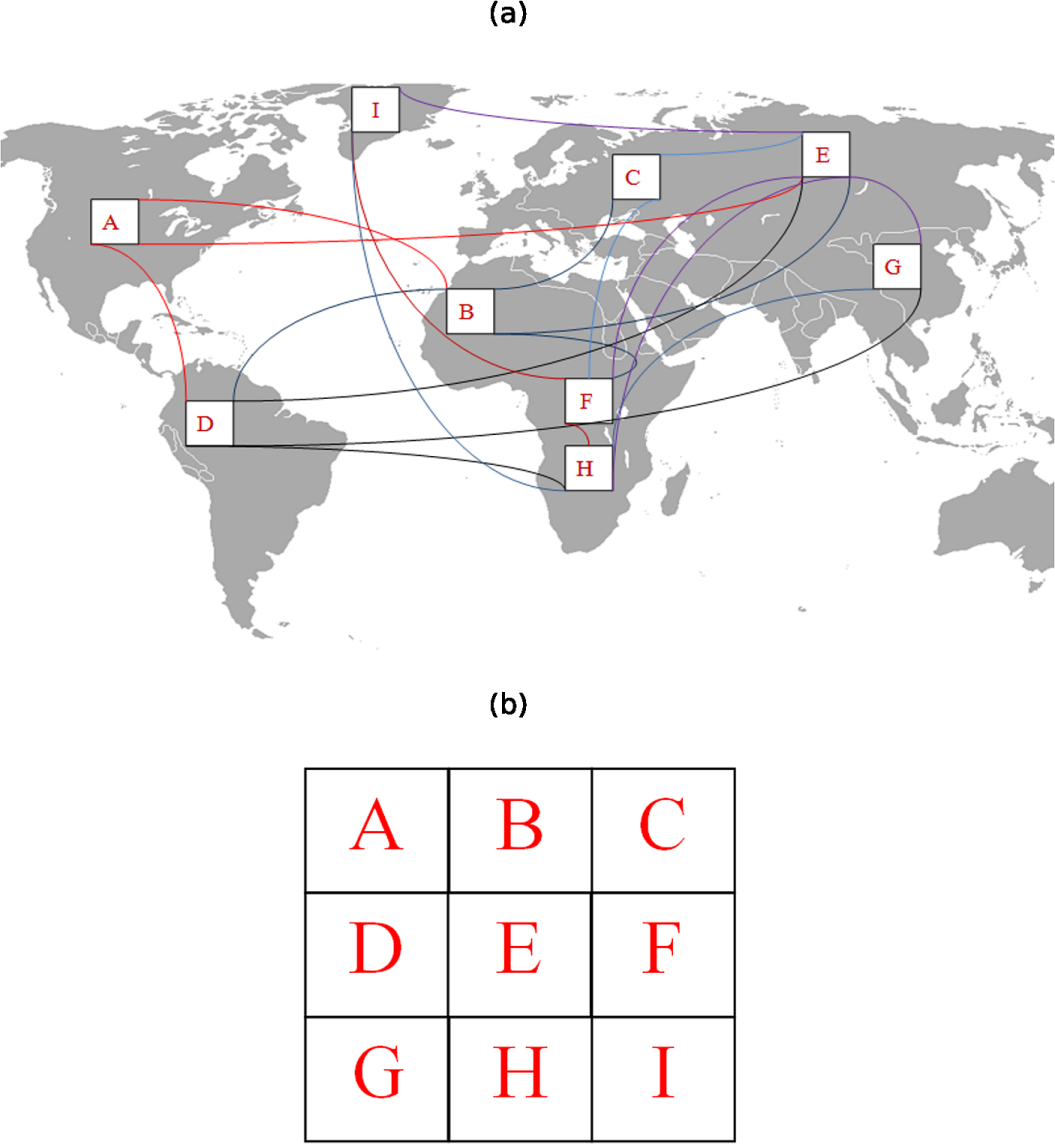
**(a): Infection travel connections between nine separated geographical regions located at different continents.** **(b)**: Assembly of the regions in one grid of nine numbered cells.

In their papers [2–4] and [5], Zakary et al. proposed new modeling and control approaches based on multi-regions discrete-time and continuous-time SIR models which have been devised in the purpose to exhibit the spatial-temporal propagation of an epidemic which emerges in different geographical regions, to show the influence which exists between regions via infection connections, and to seek a reasonable control strategy which could be effective for the prevention of infectious diseases such as HIV/AIDS and Ebola, or epidemics and pandemics in general. The authors have supposed that all regions are connected, and the infected people are able to enter all these regions. However, a region is often infected due to movements of infected people who enter from the neighboring regions. Generally, in the case of detached regions, infection travels to a targeted region if there exists a direct mode of transport between it and other regions from where the epidemic starts. In Figure 1(a), where region E is connected with all other regions, we can see an illustration of the case of infection connections, which has been studied in the references above. In the same figure, we can also see that region A is connected only with regions B, D and E. Such cases have motivated us to write this paper in order to present a new epidemic modeling approach which generalizes all possible cases of infection connections between regions. The authors in [6–8] have attempted to discuss and take into account such assumptions of connections using SIS, SIRS and SEIRS systems involving also discrete cellular simulations, but with a control approach applied to only one region.

More clearly, we propose a cellular representation of regions, assembled all together in one grid of cells, and we study the transmission dynamics of the epidemic in these regions when a travel-blocking vicinity strategy is followed for controlling one region or more to show the impact of infection connections that relate it with other regions via travel. In Figure 1(b), we can see the example of all nine regions presented in (a), how they can be converted to cells, assembled in one grid which represents a part of the earth as the global domain of interest.

Based on this new kind of representations, we can discuss the spread of the epidemic and the effectiveness of a control strategy in one region, with the possibility to analyze the SIR dynamics in this region without and with control, and exhibiting the importance of the direct influence between it and its vicinity. As observed in Figure 1(a) and (b), region I is exposed to infection via travel of infected people coming from its vicinity which contains regions D, F and H, or for the same reason, region H can directly become highly infected due to connections with regions D, E, F, G and I.

In addition to all these considerations, we note that if Zakary et al. have supposed that all regions are connected by infected travelers to show the influence of SIR dynamics of one region on other regions, the cellular model we propose here has also the advantage to exhibit this kind of influence even in the absence of direct connections between regions. This can easily be understood from the example in Figure 1 where we can see that region A can also be infected by individuals coming from region G via region D. The numerical results we will provide further are more convincing to show such kind of influence.

In the following, we provide a brief presentation of the new epidemic modeling and travel-blocking vicinity optimal control approaches.

### The new epidemic model and the vicinity travel-blocking optimal control strategy

We suggest here a new modeling approach which is based on a multi-regions discrete-time epidemic model describing the spatial-temporal spread of an epidemic which emerges in a global domain of interest Ω represented by a grid of colored cells which are uniform in size. These cells are supposed to be connected by movements of their populations, and they represent sub-domains of Ω or regions. Note that several cells are targeted by our control strategy, which means we suggest an optimization strategy that is not limited to controlling only one cell.

In [2], each region was represented by a sub-domain $(\Omega_{j})_{j=1,\ldots,p}$, while here each region or cell is denoted by $(C_{pq})_{p,q=1,\ldots,M}$.

For this, we assume that the epidemic can be transmitted and propagated by movements of people from one spatial cell $C_{pq}$ to its neighbors or cells belonging to its vicinity. In fact, in a relatively small geographical scale, some infectious diseases, such as African swine fever [9], Bovine Viral Diarrhoea virus [10, 11] and foot-and-mouth disease [12], follow that pattern of spread, and $C_{pq}$ can represent a farm; while in a large geographical scale, such as in the case of SARS [13], HIV/AIDS [4, 14], Ebola virus [5] and ZIKA virus [15], a cell $C_{pq}$ can represent a city or country. Thus, the multi-cells model with the vicinity optimal control strategy we propose here can represent good approaches for infection dynamics studies regardless of the area size. In fact, the optimization criteria are chosen in a way to restrict the movement of people coming from several cells and entering other cells. Explicitly, we seek to minimize an objective function associated with a group of cells or patch $P={ \bigcup_{p,q=1}^{m}C_{pq}}$ with $m< M$, subject to the discrete-time system associated with $C_{pq}$, with optimal controls functions introduced as effectiveness rates of the travel-blocking operations followed between *P* and its neighbors. $V_{pq}$ is the vicinity set composed of all neighboring cells of $C_{pq}$ which are denoted by $(C_{rs})_{r=p+k,s=q+k'}$ with $(k,k')\in \{-1,0,1\}^{2}$ except when $k=k'=0$. Note also, as we have mentioned before, that these cells are attached just in the grid, but in reality they are not necessarily joined together as seen in example of Figure 1(a). For instance, in Figure 1(b), the vicinity sets associated with regions or cells $C_{11}=\{A\}$, $C_{22}=\{E\}$ and $C_{32}=\{H\}$ are defined by $V_{11}=\{B,D,E\}$, $V_{22}=\{A,B,C,D,F,G,H,I\}$ and $V_{32}=\{D,E,F,G,I \}$, respectively. Thus, the travel-blocking vicinity optimal control approach will show the impact of the optimal travel-blocking control on reducing contacts between susceptible people of the targeted patch *P* and infected people coming from cells $C_{rs}$ in $V_{pq}$.

The paper is organized as follows. Section 2 presents the discrete-time multi-cells epidemic system based on a colored cell modeling approach. In Section 3, we announce a theorem of necessary conditions and characterization of the sought optimal control functions related to the travel-blocking vicinity optimal control approach. Finally, in Section 4, we provide simulations of the numerical results for an example of 100 hypothetical cities when an infection starts from one cell which has three neighboring cells (respectively, the case of a cell with eight neighboring cells is investigated), while aiming to control a patch of four cells, and in another example, two patches of one and four cells, respectively.

## A discrete-time multi-regions epidemic model

We consider a multi-regions discrete-time epidemic model which describes SIR dynamics within a global domain of interest Ω, which in turn is divided to $M^{2}$ regions, or cells, uniform in size. In other words, $\Omega ={ \bigcup_{p,q=1}^{M}C_{pq}}$ with $C_{pq}$ denoting a spatial location or region.

We note that $(C_{pq})_{p,q=1,\ldots,M}$ could represent a country, a city or a town, or a small domain such as neighborhoods, which belong respectively to the global domain of interest Ω, which could in turn represent a part of a continent or even a whole continent, a part of a country or a whole country, etc.

The S-I-R populations associated with a cell $C_{pq}$ are noted by the states $S_{i}^{C_{pq}}$, $I_{i}^{C_{pq}}$ and $R_{i}^{C_{pq}}$, and we note that the transition between them is probabilistic, with probabilities being determined by the observed characteristics of specific diseases. In addition to death, there are population movements among these three epidemiological compartments from time unit *i* to time $i+1$. We assume that the susceptible individuals are not yet infected but can be infected only through contacts with infected people from $V_{pq}$ (vicinity set or neighborhood of a cell $C_{pq}$). Thus, the infection transmission is assumed to occur between individuals present in a given cell $C_{pq}$, and it is given by

$$\sum_{C_{rs}\in V_{pq}}\beta_{rs}I_{i}^{C_{rs}}S_{i}^{C_{pq}}, $$

where $\beta_{rs}$ is the constant proportion of adequate contacts between a susceptible from a cell $C_{pq}$ and an infected coming from its neighbor cell $C_{rs}\in V_{pq}$ with $V_{pq}= \{ C_{rs}\in \Omega /r=p+k,s=q+k', ( k,k' ) \in \{ -1,0,1 \} ^{2} \} \setminus C_{pq}$.

SIR dynamics associated with a domain or cell $C_{pq}$ are described based on the following multi-cells discrete model.

For $p,q=1,\ldots,M$, we have

1 $$\begin{aligned}& S_{i+1}^{C_{pq}} = S_{i}^{C_{pq}}- \beta_{pq}I_{i}^{C_{pq}}S_{i} ^{C_{pq}}- \sum_{C_{rs}\in V_{pq}} \beta_{rs}I_{i}^{C_{rs}}S_{i}^{C_{pq}} -dS_{i}^{C_{pq}} , \end{aligned}$$

2 $$\begin{aligned}& I_{i+1}^{C_{pq}} = I_{i}^{C_{pq}}+ \beta_{pq}I_{i}^{C_{pq}}S_{i} ^{C_{pq}}+ \sum_{C_{rs}\in V_{pq}} \beta_{rs}I_{i}^{C_{rs}}S_{i}^{C_{pq}} - ( \alpha +\gamma +d ) I_{i}^{C_{pq}}, \end{aligned}$$

3 $$\begin{aligned}& R_{i+1}^{C_{pq}} = R_{i}^{C_{pq}}+\gamma I_{i}^{C_{pq}}-dR_{i} ^{C_{pq}} \end{aligned}$$

$i=0,\ldots,N-1$ with $S_{0}^{C_{pq}}\geq 0$, $I_{0}^{C_{pq}}\geq 0$ and $R_{0}^{C_{pq}} \geq 0$ being the given initial conditions.

Here, $d>0$ is the natural death rate, while $\alpha >0$ is the death rate due to the infection, $\gamma >0$ denotes the natural recovery rate from infection. By assuming that all regions are occupied by homogeneous populations, *α*, *d* and *γ* are considered to be the same for all cells of Ω.

## A travel-blocking vicinity optimal control approach

Let $I= \{ 1,2,\ldots,M \} $, $I_{H}\subset I$ be a subset of *I*, and consider $P= \{ C_{pq}/p,q\in I_{H} \} $ denoting a patch of controlled cells, with its complementary in Ω, defined as

$$\bar{P}= \{ C_{ij}/i,j\in I\backslash I_{H} \} . $$

Let us define the vicinity of the patch *P* as follows:

$$V_{P}= \{ C_{rs}\in V_{pq}\cap \bar{P}/p,q\in I_{H} \} . $$

The main goal of the travel-blocking vicinity optimal control approach is to restrict movements of infected people coming from the set $V_{P}$ and aiming to reach the patch *P* without including cells $C_{rs}$ which belong to $V_{pq}\cap P$. For this, we introduce control variables $u^{pqC_{rs}}$, which limits contacts between susceptible of the patch *P* and infected individuals from cells $C_{rs}$ that belong to $V_{P}$.

In this section, we introduce control variables in the above mentioned model to restrict contacts between susceptible people of the controlled cells $C_{pq}\in P$ and infected ones which belong to $C_{rs}\in \bar{P}\cap V_{pq}$. Then, for a given cell $C_{pq}\in \Omega $, the discrete-time system (1)-(2)-(3) becomes

4 $$\begin{aligned}& \begin{aligned}[b] S_{i+1}^{C_{pq}} = {}& S_{i}^{C_{pq}}- \beta_{pq}I_{i}^{C_{pq}}S_{i} ^{C_{pq}}- \sum_{C_{rs}\in P\cap V_{pq}} \beta_{rs}I_{i}^{C_{rs}}S_{i}^{C_{pq}} \\ & {} - \sum_{C_{rs}\in \bar{P}\cap V_{pq}} u_{i}^{pqC_{rs}} \beta_{rs}I_{i}^{C_{rs}}S_{i}^{C_{pq}}-dS_{i}^{C_{pq}}, \end{aligned} \end{aligned}$$

5 $$\begin{aligned}& \begin{aligned}[b] I_{i+1}^{C_{pq}} ={} & I_{i}^{C_{pq}}+ \beta_{pq}I_{i}^{C_{pq}}S_{i} ^{C_{pq}}+ \sum_{C_{rs}\in P\cap V_{pq}} \beta_{rs}I_{i}^{C_{rs}}S_{i}^{C_{pq}} \\ & {} + \sum_{C_{rs}\in \bar{P}\cap V_{pq}} u_{i}^{pqC_{rs}} \beta_{rs}I_{i}^{C_{rs}}S_{i}^{C_{pq}}- ( d+\alpha +\gamma ) I_{i}^{C_{pq}}, \end{aligned} \end{aligned}$$

6 $$\begin{aligned}& R_{i+1}^{C_{pq}} = R_{i}^{C_{pq}}+\gamma I_{i}^{C_{pq}}-dR_{i} ^{C_{pq}} \end{aligned}$$

$i=0,\ldots,N-1$.

Since our goal concerns the minimization of the number of infected people and the cost of the vicinity optimal control approach, we consider an optimization criterion associated with the patch *P*, and we define it by the following objective function:

7 $$\begin{aligned} \large J_{P}(u) = & \sum_{C_{pq}\in P} \Biggl[ A_{1}I_{N}^{C_{pq}}+\sum_{i=0} ^{N-1} \biggl( A_{1}I_{i}^{C_{pq}}+\sum _{C_{rs}\in \bar{P}\cap V_{pq}}\frac{A _{rs}}{2} \bigl(u_{i}^{pqC_{rs}} \bigr)^{2} \biggr) \Biggr] , \end{aligned}$$

where $A_{1}>0$ and $A_{rs}>0$ are the constant severity weights associated with the number of infected individuals and controls, respectively.

We note that here, $u= ( u_{i}^{pqC_{rs}} ) _{C_{rs}\in V_{P}, i=1,\ldots,N-1}$, which belongs to the control set $U_{P}$ defined as

$$U_{P}= \bigl\{ u/u^{\min}\leq u_{i}^{pqC_{rs}} \leq u^{\max}, i=1,\ldots,N-1, C_{rs}\in V_{P} \bigr\} . $$

Then, we seek optimal control *u* such that

$$J_{P}\bigl(u^{*}\bigr)=\min\bigl\{ J_{P}(u)/u\in U_{P}\bigr\} . $$

The sufficient conditions for the existence of optimal controls in the case of discrete-time epidemic models have been announced in [2, 3, 16] and [17].

As regards the necessary conditions and the characterization of our discrete optimal control, we use a discrete version of Pontryagin’s maximum principle [2, 3, 18]. For this, we define a Hamiltonian $\mathcal{H}$ associated with the patch *P* by

$$\begin{aligned} \mathcal{H} = & \sum_{C_{pq}\in P} \biggl[ A_{1}I_{i}^{C_{pq}}+ \sum _{C_{rs}\in \bar{P}\cap V_{pq}}\frac{A_{rs}}{2} \bigl(u_{i}^{pqC_{rs}} \bigr)^{2} \\ &{} + \zeta_{1,i+1}^{C_{pq}} \biggl( S_{i}^{C_{pq}}- \beta_{pq}I_{i}^{C _{pq}}S_{i}^{C_{pq}}- \sum_{C_{rs}\in P\cap V_{pq}}\beta_{rs}I_{i}^{C _{rs}}S_{i}^{C_{pq}} \\ &{} -\sum_{C_{rs}\in \bar{P}\cap V_{pq}}u_{i}^{pqC_{rs}} \beta _{rs}I_{i}^{C_{rs}}S_{i}^{C_{pq}}-dS_{i}^{C_{pq}} \biggr) \\ &{} + \zeta_{2,i+1}^{C_{pq}} \biggl( I_{i}^{C_{pq}}+ \beta_{pq}I_{i}^{C _{pq}}S_{i}^{C_{pq}}+ \sum_{C_{rs}\in P\cap V_{pq}}\beta_{rs}I_{i}^{C _{rs}}S_{i}^{C_{pq}} \\ & {} +\sum_{C_{rs}\in \bar{P}\cap V_{pq}}u_{i}^{pqC_{rs}} \beta _{rs}I_{i}^{C_{rs}}S_{i}^{C_{pq}}- ( d+\alpha +\gamma ) I_{i} ^{C_{pq}} \biggr) \\ &{} + \zeta_{3,i+1}^{C_{pq}} \bigl( R_{i}^{C_{pq}}+ \gamma I_{i} ^{C_{pq}}-dR_{i}^{C_{pq}} \bigr) \biggr] \end{aligned}$$

$i=0,\ldots,N-1$ with $\zeta_{k,i}^{C_{pq}}$, $k=1,2,3$, the adjoint variables associated with $S_{i}^{C_{pq}}$, $I_{i}^{C_{pq}}$ and $R_{i}^{C_{pq}}$, respectively, and defined based on formulations of the following theorem.

### Theorem 1

Necessary conditions and characterization

*Given optimal controls* $u^{pqC_{rs}*}$ *and solutions* $S^{C_{pq}^{*}}$, $I^{C_{pq}^{*}}$ *and* $R^{C_{pq}^{*}}$, *there exist* $\zeta_{k,i}^{C_{pq}}$, $i=0,\ldots,N$, $k=1,2,3$, *the adjoint variables satisfying the following equations*:

8 $$\begin{aligned}& \begin{aligned}[b] \triangle \zeta_{1,i}^{C_{pq}} = {}& {-} \biggl[ ( 1-d ) \zeta_{1,i+1} ^{C_{pq}}+ \biggl( \beta_{pq}I_{i}^{C_{pq}}+ \sum_{C_{rs}\in P\cap V_{pq}} \beta_{rs}I_{i}^{C_{rs}}+ \sum_{C_{rs}\in \bar{P}\cap V_{pq}} u_{i}^{pqC_{rs}} \beta_{rs}I_{i}^{C_{rs}} \biggr) \\ &{} \times \bigl( \zeta_{2,i+1}^{C_{pq}}-\zeta_{1,i+1}^{C_{pq}} \bigr) \biggr], \end{aligned} \end{aligned}$$

9 $$\begin{aligned}& \triangle \zeta_{2,i}^{C_{pq}} = - \bigl[ A_{1}+ \beta_{pq}S_{i}^{C _{pq}} \bigl( \zeta_{2,i+1}^{C_{pq}}- \zeta_{1,i+1}^{C_{pq}} \bigr) + ( 1-d-\alpha -\gamma ) \zeta_{2,i+1}^{C_{pq}} \bigr], \end{aligned}$$

10 $$\begin{aligned}& \triangle \zeta_{3,i}^{C_{pq}} = -(1-d)\zeta_{3,i}^{C_{pq}} \end{aligned}$$

*with* $\zeta_{1,N}^{C_{pq}}=0$, $\zeta_{2,N}^{C_{pq}}=A_{1}$, $\zeta_{3,N} ^{C_{pq}}=0$ *being the transversality conditions*. *In addition*,

11 $$\begin{aligned} u_{i}^{pqC_{rs}*} = & \min \biggl( \max \biggl( u^{\min}, \frac{(\zeta_{1,i+1} ^{C_{pq}}-\zeta_{2,i+1}^{C_{pq}})\beta_{rs}I_{i}^{C_{rs}*}S_{i}^{C _{pq}*}}{A_{rs}} \biggr),u^{\max} \biggr), \\ & i=0,\ldots,N-1,C_{rs}\in V_{P}. \end{aligned}$$

### Proof

Using a discrete version of Pontryagin’s maximum principle in [2, 3, 18], and setting $S^{C_{pq}}=S ^{C_{pq}*}$, $I^{C_{pq}}=I^{C_{pq}*}$, $R^{C_{pq}}=R^{C_{pq}*}$ and $u^{pqC_{rs}}=u^{pqC_{rs}*}$, we obtain the following adjoint equations:

$$\begin{aligned}& \triangle \zeta_{1,i}^{C_{pq}} - \frac{\partial \mathcal{H}}{ \partial S_{i}^{C_{pq}}} \\& \quad = - \biggl[ ( 1-d ) \zeta_{1,i+1}^{C_{pq}}+ \biggl( \beta_{pq}I_{i} ^{C_{pq}}+{ \sum _{C_{rs}\in P\cap V_{pq}}\beta_{rs}I_{i}^{C_{rs}}+{ \sum _{C_{rs}\in \bar{P}\cap V_{pq}}u_{i}^{pqC_{rs}} \beta_{rs}I_{i} ^{C_{rs}}}} \biggr) \\& \qquad {} \times \bigl( \zeta_{2,i+1}^{C_{pq}}-\zeta_{1,i+1}^{C_{pq}} \bigr) \biggr], \\& \begin{aligned} \triangle \zeta_{2,i}^{C_{pq}} & = - \frac{\partial \mathcal{H}}{ \partial I_{i}^{C_{pq}}} \\ & = - \bigl[ A_{1}+\beta_{pq}S_{i}^{C_{pq}} \bigl( \zeta_{2,i+1}^{C_{pq}}- \zeta_{1,i+1}^{C_{pq}} \bigr) + ( 1-d-\alpha -\gamma ) \zeta_{2,i+1}^{C_{pq}} \bigr], \end{aligned} \\& \begin{aligned} \triangle \zeta_{3,i}^{C_{pq}} & = - \frac{\partial \mathcal{H}}{ \partial R_{i}^{C_{pq}}} \\ & = -(1-d)\zeta_{3,i}^{C_{pq}} \end{aligned} \end{aligned}$$

with $\triangle \psi_{k,i}=\psi_{k,i+1}-\psi_{k,i}$, $k=1,2,3$, the difference operator, and $\zeta_{1,N}^{C_{pq}}=0$, $\zeta_{2,N}^{C_{pq}}=A _{1}$, $\zeta_{3,N}^{C_{pq}}=0$, the transversality conditions.

In order to obtain the optimality condition, we calculate the derivative of *H* with respect to $u_{i}^{pqC_{rs}}$, and we set it equal to zero

$$\frac{\partial \mathcal{H}}{\partial u_{i}^{pqC_{rs}}}=A_{rs}u_{i} ^{pqC_{rs}}- \zeta_{1,i+1}^{C_{pq}}\beta_{rs}I_{i}^{C_{rs}}S_{i}^{C _{pq}}+ \zeta_{2,i+1}^{C_{pq}}\beta_{rs}I_{i}^{C_{rs}}S_{i}^{C_{pq}}=0. $$

Then we obtain

$$u_{i}^{pqC_{rs}}=\frac{(\zeta_{1,i+1}^{C_{pq}}-\zeta_{2,i+1}^{Cpq}) \beta_{rs}I_{i}^{C_{rs}}S_{i}^{C_{pq}}}{A_{rs}}. $$

By the bounds in $U_{P}$, we finally obtain the characterization of the optimal controls $u_{i}^{pqC_{rs}^{*}}$ as

$$\begin{aligned} u_{i}^{pqC_{rs}*} = & \min \biggl( \max \biggl( u^{\min}, \frac{(\zeta_{1,i+1} ^{C_{pq}}-\zeta_{2,i+1}^{C_{pq}})\beta_{rs}I_{i}^{C_{rs}*}S_{i}^{C _{pq}*}}{A_{rs}} \biggr),u^{\max} \biggr), \\ & i=0,\ldots,N-1,C_{rs}\in V_{P}. \end{aligned}$$

 □

## Numerical results and discussions

### Brief presentation

In this section, we provide numerical simulations to demonstrate our theoretical results in the case when the studied domain Ω represents the assembly of $M^{2}$ regions or cells (cities, towns, …). A code is written and compiled in MATLAB using data cited in Table 1. The optimality systems are solved using an iterative method where at instant *i* the states $S_{i}^{C_{pq}}$, $I_{i}^{C_{pq}}$ and $R_{i}^{C_{pq}}$ with an initial guess are obtained based on a progressive scheme in time, and their adjoint variables $\zeta_{l,i} ^{C_{pq}}$, $l=1,2,3$, are obtained based on a regressive scheme in time because of the transversality conditions. Afterwards, we update the optimal control values (11) using the values of state and costate variables obtained in the previous steps. Finally, we execute the previous steps till a tolerance criterion is reached. In order to show the importance of our work, and without loss of generality, we consider here that $M=10$, and then we present our numerical simulations in a $10\times 10$ grid which represents the global domain of interest Ω.

**Table 1:** **Parameter values of** ***α*** **,** ***β*** **,** ***γ*** **and** ***d*** **associated with a cell** $\pmb{C_{pq}}$ **,** $\pmb{p,q=1,\ldots,M}$ **, utilized for the resolution of all multi-regions discrete-time systems (1)-(3) and (4)-(6), and then leading to simulations obtained from Figure** **2** **to Figure** **19** **, with the initial conditions** $\pmb{S_{0}^{C_{pq}}}$ **,** $\pmb{I_{0}^{C_{pq}}}$ **and** $\pmb{R_{0}^{C_{pq}}}$ **associated with any cell** $\pmb{C_{pq}}$ **of** **Ω**

$\boldsymbol{S_{0}^{C_{pq}}}$	$\boldsymbol{I_{0}^{C_{pq}}}$	$\boldsymbol{R_{0}^{C_{pq}}}$	***α***	***β***	***γ***	**d**
50	0	0	0.002	0.0001	0.003	0.0001

At the initial instant $i=0$, susceptible people are homogeneously distributed with 50 individuals in each cell except at the lower right corner cell $C_{11}$, where we introduce 10 infected individuals and 40 susceptible ones. With similar values, we study the case when the epidemic starts from cell $C_{65}$ which is near to the center of Ω.

In all of the figures, the redder part of the color-bars contains larger numbers of individuals, while the bluer part contains smaller numbers. In the following, we discuss in more detail the cellular simulations we obtain in the case when there is yet no control.

### Cellular simulations without controls

**Figure 2:**
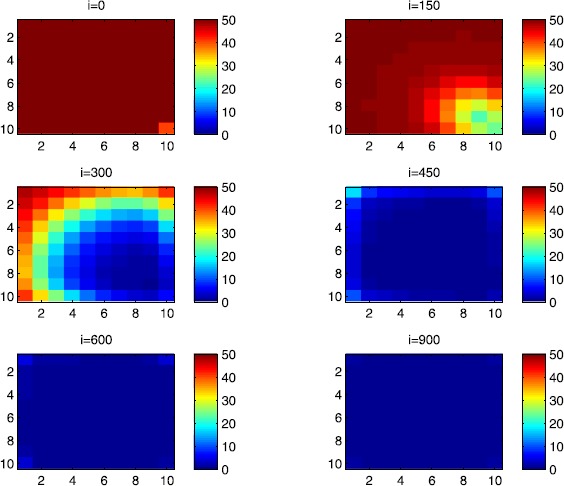
$\pmb{S^{C_{pq}}}$ **behavior in the absence of control.** The case when the disease starts from the corner $C_{11}$.

**Figure 3:**
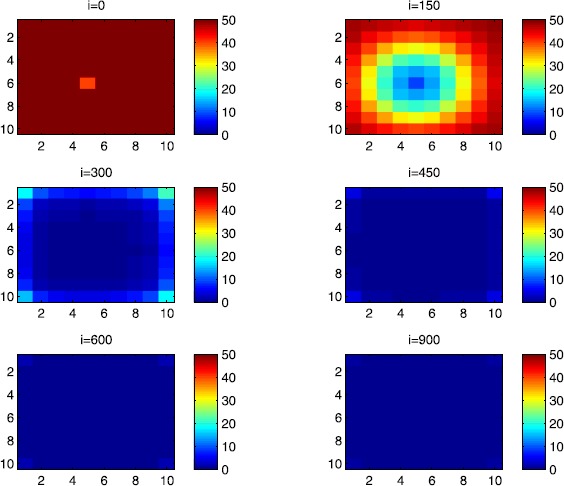
$\pmb{S^{C_{pq}}}$ **behavior in the absence of control.** The case when the disease starts from the center of Ω.

**Figure 4:**
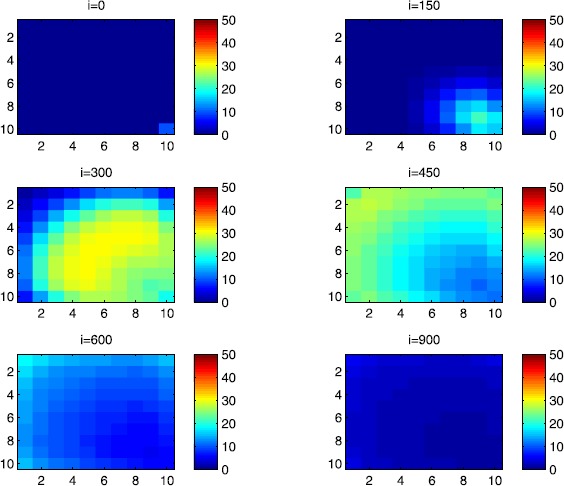
$\pmb{I^{C_{pq}}}$ **behavior in the absence of control.** The case when the disease starts from the corner $C_{11}$.

**Figure 5:**
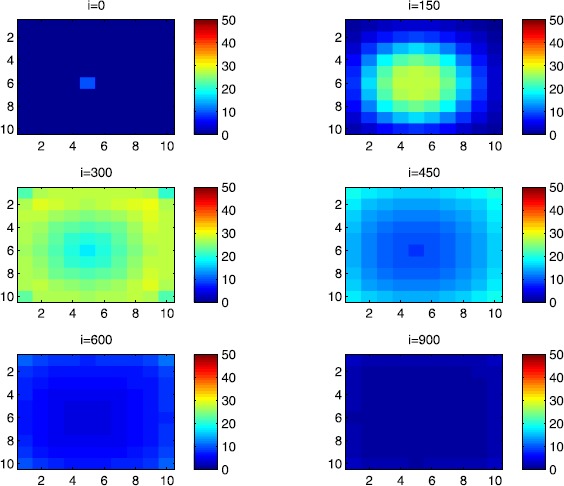
$\pmb{I^{C_{pq}}}$ **behavior in the absence of control.** The case when the disease starts from the center of Ω.

**Figure 6:**
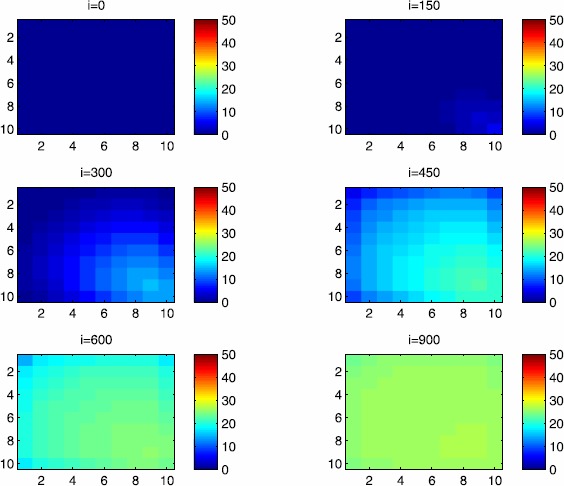
$\pmb{R^{C_{pq}}}$ **behavior in the absence of control.** The case when the disease starts from the corner $C_{11}$.

**Figure 7:**
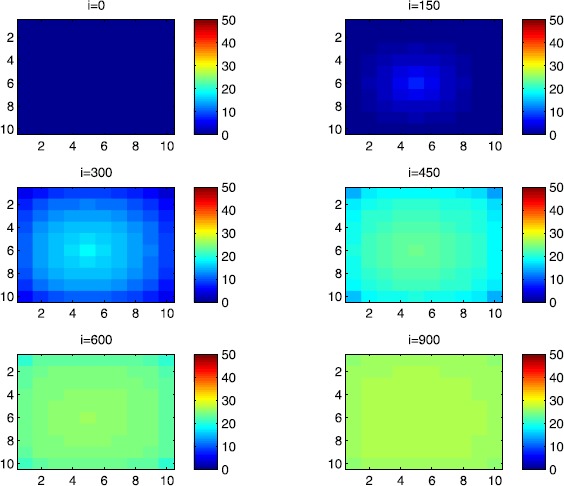
$\pmb{R^{C_{pq}}}$ **behavior in the absence of control.** The case when the disease starts from the center of Ω.

In this section, Figures 2, 3, 4, 5, 6 and 7 depict dynamics of the susceptible population in the case when there is yet no control strategy to be followed for the prevention of the epidemic. We note that in all the figures presented here, simulations give us an idea about the spread of the disease in two different cases:

- when the epidemic starts in a cell $C_{pq}$ with $p=10$, $q=10$ (lower right corner cell). It represents the case when the vicinity set $V_{pq}$ associated with the source cell of infection contains three cells.
- when the epidemic starts from a cell $C_{pq}$, with $p=6$, $q=5$, located in the vicinity of the target patch we aim to control.

For instance, in Figure 2, if we suppose there are 40 susceptible people in cell $C_{1010}$ located at the lower right corner of Ω, and 50 in each of the other cells, we can see that at instant $i=150$, the number $S^{C_{1010}}$ becomes less important and takes a value close/or equal to 20, while $S^{C_{pq}}$ in the cells of $V_{1010}$ takes values close/or equal to 30. As we move away from $V_{1010}=\{C_{109},C_{910},C _{99}\}$, $S^{C_{pq}}$ remains important. At instant $i=300$, we can observe that in most of cells $S^{C_{pq}}$ becomes less important, taking values between 0 and 10, while in other cells it takes values between 20 and 40 except $S^{C_{1010}}$ which conserves its value in 50 since it is located far away from the source of infection. At instant $i=450$, $S^{C_{pq}}$ becomes zero except at the corners and in most cells at the borders of Ω, because these cells have vicinity sets smaller than other cells. Finally, at last instants, $S^{C_{pq}}$ converges to zero in all cells. As regards Figure 3, when we consider $S^{C_{65}}=40$, which is located near the center of Ω, and 50 susceptible people in each of the other cells, it is observed that the situation is more severe, because the disease reaches the corners and borders faster than in the case of Figure 2. As we can see, at instant $i=300$, $S^{C_{pq}}$ takes values less important in most cells except at the corners and borders since their vicinity sets contain only three to five cells respectively, but it is the result we have reached until instant $i=450$ in Figure 2.

Figures 4 and 5 illustrate the rapid propagation of the infection when the disease starts from cell $C_{1010}$ and from the center of Ω, respectively. In Figure 4, if we suppose there are ten infected people in cell $C_{1010}$ and no infection in all other cells, we observe that at instant $i=150$ the number $I^{C_{1010}}$ increases to bigger values close/or equal to 30 in $C_{99}$, while $I^{C_{pq}}$ in the cells of $V_{1010}$ takes values close/or equal to 20, and as we move away from $V_{1010}$, $I^{C_{pq}}$ remains less important. At instant $i=300$, we can see that in most of cells, $I^{C_{pq}}$ becomes more important, taking values between 30 and 35 in the cells which are close to the cells with eight neighboring cells, while in few other cells, it takes values between 0 and 20. From these numerical results, we can deduce that once the infection arrives to the center or to the cells with eight cells in their vicinity sets, the infection becomes more important compared to the case of the previous instant. At instant $i=450$, $I^{C_{pq}}$ takes values close/or equal to 20 in the cell from where the epidemic has started, and 25 in $V_{1010}$ and near to it, and as we move towards the center and further regions, infection is important with the presence of more than 30 infected individuals in each cell except the ones in the three opposite corners even at instant $i=600$. In fact, at the center of Ω, the number of infected people, which has increased to 35 at the previous instant, has been reduced, because once a cell becomes highly infected, it loses an important number of individuals which die or recover naturally after. All cells $C_{pq}$ become highly infected and the number $I^{C_{pq}}$ becomes less and less important at further instants, noting that at $i=900$, a large number of infected individuals has decreased because many $I^{C_{pq}}$ have died or moved to the removed compartment. In Figure 5, when we consider infection starting from near the center of Ω by considering now that $I^{C_{65}}=10$, and no infected people in other cells, the disease spreads towards the corners and borders faster than in the first case in Figure 4. At instant $i=150$, the number of infected people has increased in $V_{65}$, and as we move away to the corners and borders, infection is still low. At instant $i=300$, $I^{C_{pq}}$ takes values more important in most cells, close/or equal to 35, except at the corners and center where $I^{C_{pq}}$ is close/or equal to 30, which is the result we can reach until instant $i=450$ in Figure 4, noting that $I^{C_{65}}$ and $I^{C_{pq}}$ in $V^{C_{65}}$ have reduced due to death or natural recovery from the disease, while the infection has remained important in the cells which are near to the corners and borders since the infection has just arrived. The corner cells at instant $i=450$ conserve their number of infected individuals, while cells at the borders of Ω and the ones which are close to the center lose more people due to the number of dead or recovered people, which increases more and more at further instants, leading $I^{C_{65}}$ to decrease towards 13 and 8 at $i=600$ and $i=900$, respectively.

We note that in the following figure, the scale of the color-bars does not exceed ten individuals since we cannot reach a larger number of removed people when we focus only on targeting infected people which come from $C_{1010}$. As we can observe in Figure 6, when we have supposed there are 40 susceptible people in cell $C_{1010}$, and 50 in each of the other cells, we can see here that simultaneously, at instant $i=150$, the numbers $R^{C_{1010}}$ and $R^{C_{pq}}$ in the cells of $V_{11}$ are close/or equal to only one or two removed people, and as we move away from $V_{1010}$, $R^{C_{pq}}$ becomes zero. Similarly, at instant $i=300$, the number $R^{C_{pq}}$ is not zero and takes values between one and three, except for distant cells where it remains zero. At instant $i=450$, $R^{C_{pq}}$ takes values between three and five except at the opposite three corners and some cells at the borders where it does not exceed two removed people. Finally, at further instants $R^{C_{pq}}$ converges to five in most cells at $i=600$ and in all cells at $i=900$ since as we go forward in time, some people acquire immune responses that help them to cure naturally from the disease. As regards Figure 7, when we consider $S^{C_{65}}=40$ located near the center of Ω, we can see that the results at instant $i=600$ when the disease has started from the upper left corner of Ω are at most the same as the results obtained at $i=450$ when the disease has started near the center of Ω. At instant $i=300$, $R^{C_{pq}}$ has already begun to increase from the center because some infected people have disappeared as seen in the previous figure. As regards further numerical simulations, we can observe that the number of the removed people increases to five at the center at $i=450$ until it reaches the same value in all cells of Ω except the corners at $i=600$. It becomes more and more important at further instants reaching five removed people at the corners and six in each of the other cells at $i=900$.

In the following, we discuss the cellular simulations we obtain in the case when the optimal controls (11) are introduced.

### Cellular simulations with controls

**Figure 8:**
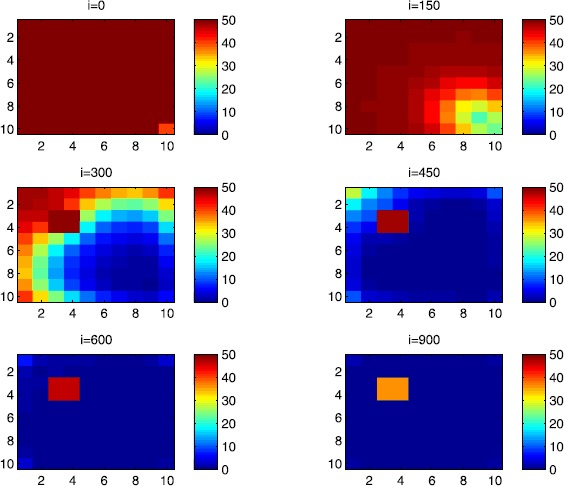
$\pmb{S^{C_{pq}}}$ **behavior in the presence of optimal controls (** **11** **).** The case when the disease starts from the corner $C_{11}$.

**Figure 9:**
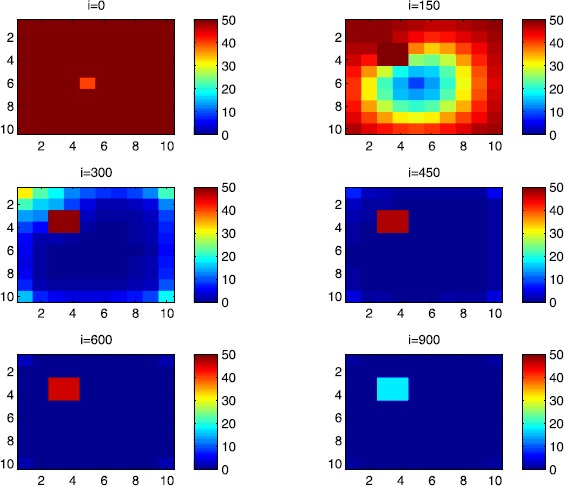
$\pmb{S^{C_{pq}}}$ **behavior in the presence of optimal controls (** **11** **).** The case when the disease starts from the center of Ω.

**Figure 10:**
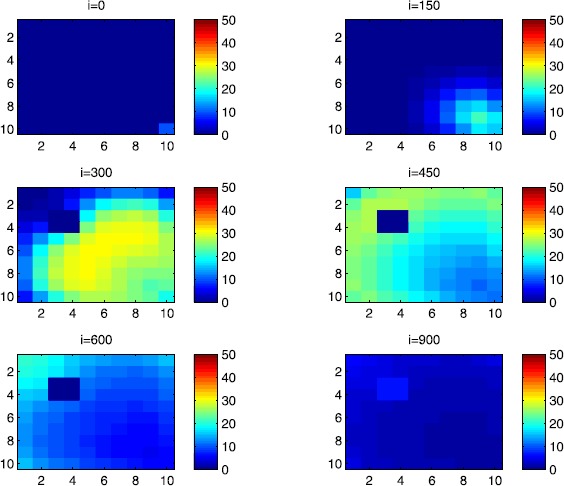
$\pmb{I^{C_{pq}}}$ **behavior in the presence of optimal controls (** **11** **).** The case when the disease starts from the corner $C_{11}$.

**Figure 11:**
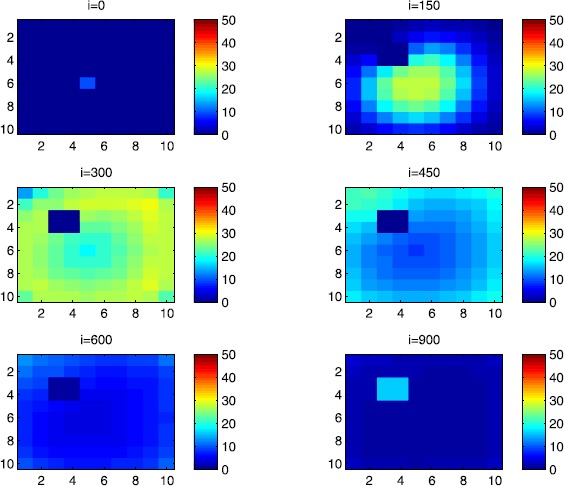
$\pmb{I^{C_{pq}}}$ **behavior in the presence of optimal controls (** **11** **).** The case when the disease starts from the center of Ω.

**Figure 12:**
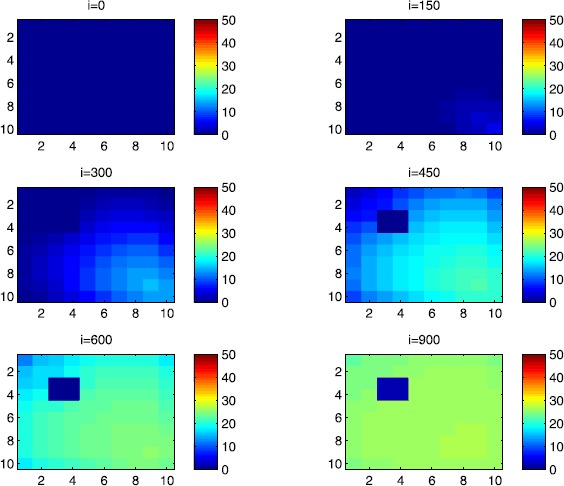
$\pmb{R^{C_{pq}}}$ **behavior in the presence of optimal controls (** **11** **).** The case when the disease starts from the corner $C_{11}$.

**Figure 13:**
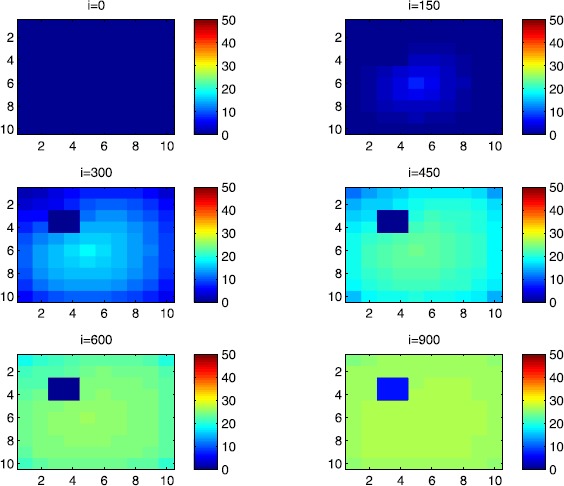
$\pmb{R^{C_{pq}}}$ **behavior in the presence of optimal controls characterized in (** **11** **).** The case when the disease starts from the center of Ω.

Figures 8, 9, 10, 11, 12 and 13 depict dynamics of the SIR populations when the travel-blocking vicinity optimal control strategy is followed.

In order to show the importance of the optimal control approach suggested in this paper, we take the example of a patch which has 12 neighboring cells. As it was done in the previous part, we investigate also here the results obtained when the disease starts from a corner and when it starts near or attached to the center. As an example, we suppose that the patch we aim to control is $P=\{C_{33},C_{34},C_{43},C_{44} \}$, and we present simulations when the epidemic is more important at the corner cell $C_{1010}$ and when the epidemic is more important in cell $C_{65}$ which is attached or directly connected to *P*.

In the following, we consider that the vicinity of the patch *P* is defined by

$$V_{P}=\{C_{22},C_{23},C_{24},C_{25},C_{32},C_{35},C_{42},C _{45},C_{52},C_{53},C_{54},C_{55} \}. $$

In Figure 8, as supposed also above, there are 40 susceptible people in cell $C_{1010}$, and 50 in each of the other cells. We can see that at instant $i=150$, the numbers $S^{C_{1010}}$ and $S^{C_{pq}}$ are at most the same as in the case when there was no control strategy. At instant $i=300$, we can observe that in most of cells, $S^{C_{pq}}$ becomes less important, taking values between zero and ten in cells that are close to $V_{1010}$, while in other cells, and as we move away from $V_{1010}$, it takes values between 20 and 40. However, the controlled patch *P* contains 50 susceptible people in each cell. In fact, even at instant $i=150$, the number of susceptible people in the controlled patch conserved its value in 50, which is not also exactly the same as in the case when there was yet no control strategy since in Figure 2, $S^{C_{pq}}$ has decreased more significantly. Thus, we can deduce that the travel-blocking vicinity optimal control strategy has proved its effectiveness earlier in time. At instants $i=450,600$ and $i=900$, $S^{C_{pq}}$ is also the same as done before but fortunately again, we reach our goal in keeping the number of susceptible people in *P* close to its initial value despite a decrease of 15 people. Thus, this demonstrates that most of movements of infected people coming from the vicinity of *P* have been restricted in final times. As regards Figure 9, we consider $S^{C_{65}}=40$ which represents the number of susceptible people present in cell $C_{65}$ located near the center of Ω which is more close to the target cell *P*, and we consider 50 susceptible people in each of the other cells. We can observe at instant $i=300$ that $S^{C_{pq}}$ takes values less important in most cells except at the corners and borders since the number of cells in their vicinity sets is small, but it is the result we have reached until instant $i=450$ in Figure 8. We can see more clearly that the number of susceptible people in the patch *P* has decreased to only 45 in each cell $C_{pq}\in P$, since even in the previous instant $i=150$, $S^{C_{55}}$ has not changed also and conserved its value in 50. At that instant, and as shown also in the previous case, the corner cells contain about 20 susceptible, while in cells in the borders, $S^{C_{pq}}$ takes values between 10 and 15, and 0 in some cells near to the corners. Finally, as we move forward in time, it is observed that the number of susceptible people in the targeted patch *P*, in each cell $C_{pq}\in P$, has decreased to 20, which is smaller than the number of susceptible people in the case when the infection has started from the corner. In fact, it is an interesting result since it shows the impact of infection which starts close to the targeted patch even if it does not start exactly in the vicinity of the patch *P*.

In Figure 10, we can see more the analogy between the number of susceptible people $S^{C_{1010}}$ and $S^{C_{65}}$ and infected ones $I^{C_{11}}$ and $I^{C_{65}}$. In fact, when the disease starts from cell $C_{1010}$, as supposed in the section above, there are ten infected people in cell $C_{1010}$ and no infected in each of the other cells. We can deduce that at instant $i=150$, the numbers $I^{C_{1010}}$ and $I^{C_{pq}}$ are at most the same, as shown in the absence of controls. At instant $i=300$, we can see that in most of cells, $I^{C_{pq}}$ is similar to the case in Figure 3, and it is also more important, taking values between 20 and 30; while in other cells, it takes values between 0 and 10 as shown in the previous subsection. However, the controlled patch *P* is still not really infected since it does not contain yet any infected individual. At instant $i=450$, $I^{C_{pq}}$ takes values around 20 in neighboring cells which belong to $V_{1010}$, and about 30 in other cells except at the three opposite corners and borders of Ω. At instant $i=600$, most cells $C_{pq}$ begin to lose some infected individuals due to natural recovery, and the number $I^{C_{pq}}$ becomes less and less important at further instants, while the number of infected people in the patch *P* does not exceed eight infected individuals. In Figure 11, and as done in the case without controls, we can also observe that when we consider infection starting from near the center of Ω by supposing ten infected individuals in each cell of the patch *P* with no infected people in each of the other cells outside the patch, the disease spreads towards the corners and borders faster than in the first case in Figure 10. For instance, at instant $i=150$, the number of infected people has increased in the vicinity of the patch *P* and in $V_{65}$, and as we move away to the corners and borders, infection is still low. At instant $i=300$, $I^{C_{pq}}$ takes values more important in most cells except at the corners, which is the result we can reach until instant $i=600$ in Figure 10, noting that the number of infected people in the patch *P*, and $I^{C_{pq}}$ in the vicinity of the patch *P*, has reduced due to death or natural recovery from the disease, while the infection becomes important in cells which are near to the corners and borders. The corner cells at instant $i=450$ conserve their number of infected individuals while some cells at the borders of Ω and the ones which are close to the center lose more people due to the number of dead or removed people, which increases more and more at further instants as observed in $i=600$ and $i=900$ where the number of infected people in *P* does not exceed 20 infected individuals, which is bigger than the number of infected people in the case when the infection has started from the corner. Thus, as deduced in Figure 9, it also shows the impact of infection which starts close to the targeted patch even if it does not start exactly in the vicinity of the patch *P*.

In Figure 12, when we suppose there are 40 susceptible people in cell $C_{1010}$, and 50 others in each of the other cells, we can see that simultaneously, at instant $i=150$, the number $R^{C_{1010}}$ takes a value close/or equal to five, while $R^{C_{pq}}$ in cells of $V_{1010}$ are zero, and as we move away from $V_{1010}$, $R^{C_{pq}}$ is still zero. Similarly, at instant $i=300$, the number $R^{C_{pq}}$ is zero at the three opposite corners and borders of Ω, while it takes values between 10 and 20 in other cells, but the number of removed people in the patch *P* is still very close to zero due to very few people who have been infected there. At instant $i=450$, $R^{C_{pq}}$ takes values between 15 and 20 except at the corners and borders, while *P* is still not containing any individual in its removed compartment. Finally, at last instants, $R^{C_{pq}}$ converges to 20 at $i=600$ in all cells except in *P*, which does not exceed three removed people, and between 25 in all cells at $i=900$ and a number of individuals close to five in *P* since not many individuals have been infected to move to the removed compartment. As regards Figure 13, when we consider $S^{C_{65}}=40$, we can see that at instant $i=600$, in all cells $C_{pq}$, the number of the removed people increases to 20 in the corner cells, and 25 in most cells of Ω, and to 25 when we go forward in time as we can observe at instant $i=900$, while the number of removed people in the patch *P* has not exceeded about eight removed people.

### Discussions

**Figure 14:**
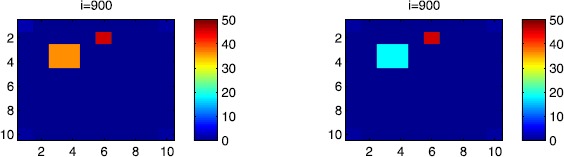
$\pmb{S^{C_{pq}}}$ **behavior at the final instant** $\pmb{i=900}$ **in the presence of optimal controls characterized in (** **11** **) with a travel-blocking vicinity optimal control strategy followed in the two patches** ***P*** **and** $\pmb{P'=\{C_{26}\}}$ **.** On the left side, the case when the disease starts from the corner cell $C_{1010}$. On the right side, the case when the disease starts near the center of Ω, exactly at $C_{65}$.

**Figure 15:**
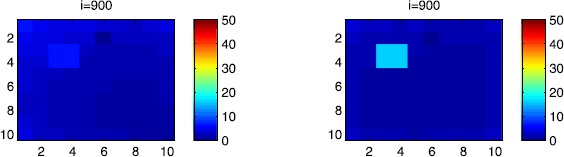
$\pmb{I^{C_{pq}}}$ **behavior at the final instant** $\pmb{i=900}$ **in the presence of optimal controls characterized in (** **11** **) with a travel-blocking vicinity optimal control strategy followed in the two patches** ***P*** **and** $\pmb{P'=\{C_{26}\}}$ **.** On the left side, the case when the disease starts from the corner cell $C_{1010}$. On the right side, the case when the disease starts near the center of Ω, exactly at $C_{65}$.

**Figure 16:**
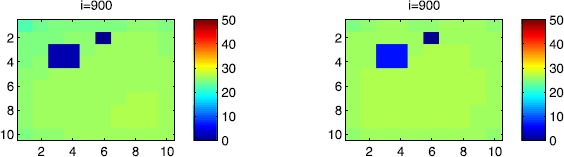
$\pmb{R^{C_{pq}}}$ **behavior at the final instant** $\pmb{i=900}$ **in the presence of optimal controls characterized in (** **11** **) with a travel-blocking vicinity optimal control strategy followed in the two patches** ***P*** **and** $\pmb{P'=\{C_{26}\}}$ **.** On the left side, the case when the disease starts from the corner cell $C_{1010}$. On the right side, the case when the disease starts near the center of Ω, exactly at $C_{65}$.

In Figures 14, 15 and 16, we investigate the effectiveness of the travel-blocking vicinity optimal control approach on the SIR populations of Ω when it is applied to two patches *P* and $P'=\{C_{26}\}$. We can see that at instant $i=900$ the number of SIR people in the patch *P* is the same as shown in Figures 8, 9, 10, 11, 12 and 13 regardless of the source of infection (from the corner cell $C_{1010}$ or from $C_{65}$). However, the most interesting idea we can extract from this figure is that regardless of the source of infection, the number of susceptible people in the patch $P'$ has not decreased significantly. When we aim to control only one cell, the vicinity set associated with this cell contains eight cells, while in the patch *P* considered here, each cell $C_{pq}\in P$ is under control with a travel-blocking strategy applied to only five cells in its vicinity. Consequently, the movements of infected travelers entering from the three remaining obvious neighboring cells are not restricted. For instance, in Figure 14, the number of susceptible people in $P'$ is equal to 45 on both sides, which means it has decreased by only five people from its initial condition. On the other hand, in the patch *P*, the number of susceptible people has decreased by more than 15 and 30 people from the initial conditions when the epidemic starts from the corner cell $C_{1010}$ and $C_{65}$, respectively. Also, in Figure 15, the number of infected people in $P'$ is equal to zero on both sides, which means it has not increased at all; while in the patch *P*, the number of infected people has increased by more than 5 and 20 people from the initial conditions when the epidemic starts from the corner and near the center of Ω, respectively. Finally, in Figure 16, we can observe on both sides that the number of removed people has not increased at all in $P'$ since there was no real infection; while in the patch *P* the number of removed people has increased by two to five individuals from the corner and near the center of Ω, respectively.

**Figure 17:**
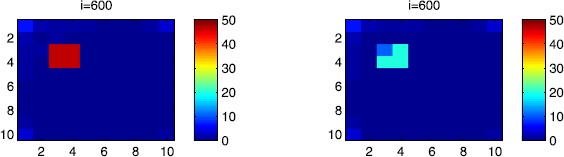
$\pmb{S^{C_{pq}}}$ **behavior at instant** $\pmb{i=600}$ **in the presence of optimal controls characterized in (** **11** **).** On the left side, the case when the travel-blocking vicinity control approach is applied to all cells $C_{pq}\in P$. On the right side, the case when the travel-blocking vicinity optimal control strategy is followed in $P\setminus C_{33}$. The infection starts from the lower left corner cell $C_{1010}$.

**Figure 18:**
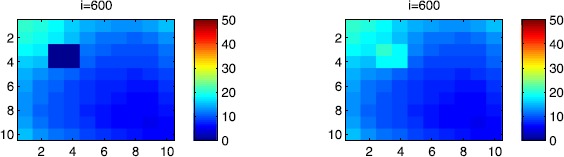
$\pmb{I^{C_{pq}}}$ **behavior at instant** $\pmb{i=600}$ **in the presence of optimal controls characterized in (** **11** **).** On the left side, the case when the travel-blocking vicinity control approach is applied to all cells $C_{pq}\in P$. On the right side, the case when the travel-blocking vicinity optimal control strategy is followed in $P\setminus C_{33}$. The infection starts from the lower left corner cell $C_{1010}$.

**Figure 19:**
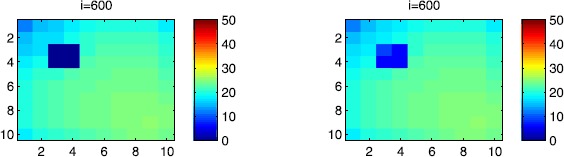
$\pmb{R^{C_{pq}}}$ **behavior at instant** $\pmb{i=600}$ **in the presence of optimal controls characterized in (** **11** **).** On the left side, the case when the travel-blocking vicinity control approach is applied to all cells $C_{pq}\in P$. On the right side, the case when the travel-blocking vicinity optimal control strategy followed in $P\setminus C_{33}$. The infection starts from the lower left corner cell $C_{1010}$.

In Figures 17, 18 and 19, we illustrate SIR dynamics in Ω at instant $i=600$ by giving a comparison between two cases:

- when the travel-blocking vicinity optimal control is applied to all cells $C_{pq}$ which belong to the patch *P*.
- when the travel-blocking vicinity optimal control is applied to all cells $C_{pq}$ which belong to the patch *P*, except in $C_{33}$.

The cellular simulations on the right side are associated with the first case, while the other ones on the left side are associated with the second case.

As we can observe in Figure 17, on the left side, the number of susceptible people in $C_{pq}\in P$ has not changed significantly compared to the initial conditions. It loses now more people as seen in the cellular simulations on the right side. Even when we consider an infection which starts from the left lower corner cell $C_{1010}$ and the travel-blocking vicinity optimal control strategy is considered to be missed in only one cell $C_{33}$, the number of susceptible people in $P\setminus C_{33}$ has decreased to smaller values which equal 20 individuals in each cell. Moreover, obviously, cell $C_{33}$ loses more susceptible people towards 10, which is due to the movements of infected people that were not restricted in $V_{33}$. As regards the cellular simulations in Figure 18, we can see in cellular simulations on the left side that when the travel-blocking vicinity optimal control approach was followed in all cells $C_{pq}\in P$, the number of infected people has not increased and conserved the zero value, while in cellular simulations on the right side, the number of infected people has increased to 20 in each cell in $P\setminus C_{33}$ and to 25 in $C_{33}$. Simultaneously, we can see in Figure 19 that the number of the removed people in cellular simulations on the left side has not increased since there was no real infection after applying the travel-blocking vicinity optimal control strategy in all cells $C_{pq}\in P$. However, when we do not restrict movements of infected people coming from $V_{33}$, we can see that the number of removed people has increased to five and ten individuals in $P\setminus C_{33}$ and $C_{33}$, respectively. All that means that if the infection started from the upper right corner cell, the situation would be more severe. Also, this comparison shows the importance and utility of the application of the travel-blocking vicinity optimal control strategy in all cells which belong to the vicinity set $V_{P}$ since the infection which comes from only one cell could lead to undesirable results.

**Figure 20:**
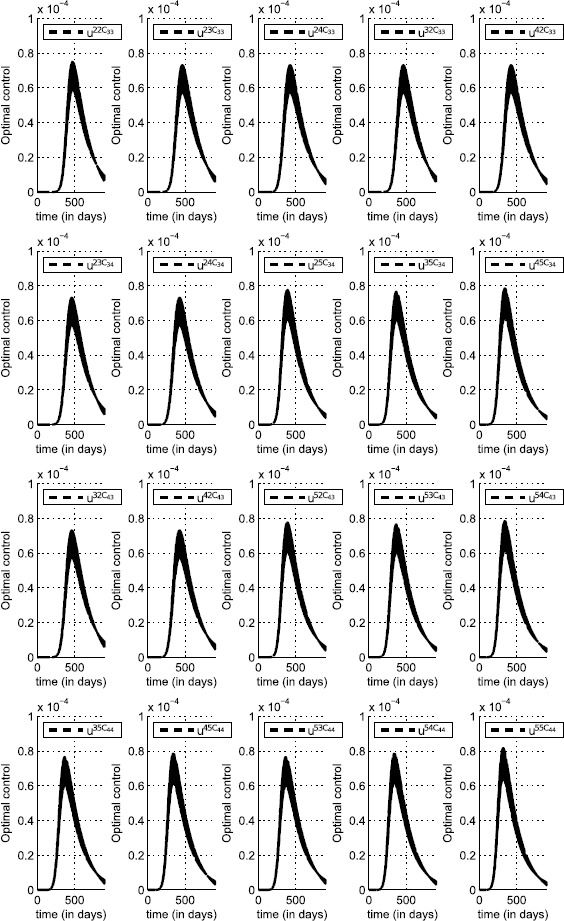
**The optimal controls (** **11** **) associated with each cell in** ***P*** **(i.e.,** $\pmb{C_{33}}$ **,** $\pmb{C_{34}}$ **,** $\pmb{C_{43}}$ **and** $\pmb{C_{44}}$ **).**

**Figure 21:**
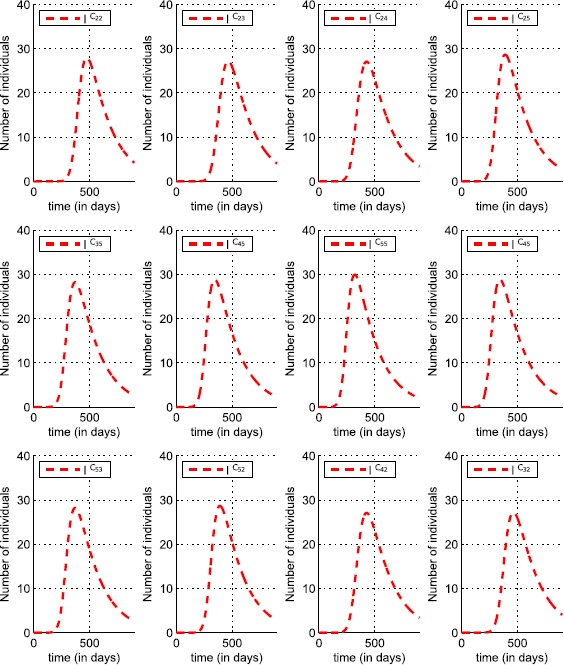
$\pmb{I^{C_{rs}}}$ **behavior in the vicinity set** $\pmb{V_{P}}$ **, with** $\pmb{C_{rs}\in V_{P}}$ **.**

In Figure 20, we can see the shapes of the optimal controls associated with cells $C_{33}$, $C_{34}$, $C_{43}$ and $C_{44}$ which belong to the patch *P*. Since each cell in Ω that is not located at the corners and borders has eight neighboring cells, based on the location of the patch *P* we consider here, each cell in *P* has also eight cells in its vicinity. However, the travel-blocking vicinity optimal control strategy is proposed to be applied exactly to the patch *P* or to all cells in *P* together, and not to each cell alone. In fact, we may not suggest a control strategy which aims to restrict movements of people traveling from one cell to another cell belonging to the same patch. It would lead to saying that in each cell in *P* we should not isolate people without letting them reach other cells which belong to the same patch. However, in terms of the costs of an optimization approach, it would be more beneficial to control a patch rather than controlling cell by cell in the patch. Thus, the neighboring cells in *P* associated with a cell $C_{pq}\in P$ are not subject to any movement restriction, which means the number of optimal controls that have to be introduced in the vicinity of a cell $C_{pq}\in P$ is five rather than eight since the three neighboring cells which belong to *P* are missed. Then, for the patch *P* containing four cells, we have 20 optimal controls as we can observe in Figure 19. The optimal controls share similar shapes where most of their curves vary from 0 to $0.82\times 10^{-4}$. The values of the optimal controls are small but realize our main objective presented in (7), but the most important idea we can extract from the results in both Figures 20 and 21 is that there is an analogy between shapes of infection and optimal control functions since whenever the infection is maximal, the optimal controls which are associated with it become simultaneously maximal. Respectively, this remains true when the infection is minimal as we can see at final times. In fact, even under the initial conditions, there is an analogy between $u^{pqC_{rs}}$ and $I^{C_{rs}}$. This means that once an infection is detected, maximized or minimized in the vicinity of the targeted cell, the travel-blocking optimal control function responds automatically and similarly at the same time.

## Conclusion

Some researchers have exploited the framework of compartmental modeling in epidemiology and tried to introduce the concept of networks-based models either for the description of social contagion processes as done in [19] or for the study of the propagation of electronic and computer viruses as in [20, 21]. Not very far from the main goals of this kind of epidemic models treated in the mentioned references, which aim to highlight the nature of infection connections which participate in the rapid spread of an epidemic, in this paper we have devised a multi-regions discrete-time model which describes infection dynamics due to the presence of an epidemic in one region and its spreading to other regions via travel. Regions have been assembled in one grid of cells, where each cell represents a region, in order to exhibit the impact of infection which comes from the vicinity of a patch. In fact, by this kind of representations, we have succeeded to show the effectiveness of the travel-blocking vicinity optimal control approach when it is applied to patches. Then, we demonstrated that when we restrict movements of infected people coming from the vicinity of a targeted patch, we can keep this patch safe without or with important infection.

These modeling and optimal control approaches have also led us to three major results:

- when an epidemic starts near the center of a global domain of interest, the situation becomes more severe in terms of the number of infected individuals compared to the case when the epidemic starts from a corner. This is due to the number of cells in the vicinity of the cell that represents the source of infection.
- the optimal controls introduced in our mathematical model respond automatically to the epidemic once it is detected, and there is an analogy between their shapes and the shape of infection.
- if we do not apply the travel-blocking vicinity optimal control strategy to only one cell of the targeted patch, the other optimal controls are not sufficient to stop or to reduce the infection in the controlled patch.

The cellular simulations we presented in the numerical results section have illustrated the case of 100 cells threatened by infection coming from one cell first located in the corner of a global domain of interest, and then near the center of this domain, while the patch targeted for control was chosen to contain four cells located near the center.
